# Bananas in the aftermath of La Palma volcanic eruption (Canary Islands, Spain): A study on the nutritional and toxic element composition of post-disaster production

**DOI:** 10.1371/journal.pone.0328982

**Published:** 2025-08-11

**Authors:** Ángel Rodríguez-Hernández, Norberto Ruiz-Suárez, Luis Alberto Henríquez-Hernández, Ricardo Díaz-Díaz, Manuel Zumbado, María del Mar Bernal Suárez, Pablo Alonso-González, Eva Parga-Dans, Octavio P. Luzardo

**Affiliations:** 1 Toxicology Unit, Department of Biomedical Sciences, Universidad de Alcalá, Carretera Madrid-Barcelona, Spain; 2 Toxicology Unit, Research Institute of Biomedical and Health Sciences (IUIBS), Universidad de Las Palmas de Gran Canaria, Las Palmas de Gran Canaria, Spain; 3 Spanish Biomedical Research Center in Physiopathology of Obesity and Nutrition (CIBERObn), Spain; 4 Department of Environmental Analysis, Instituto Tecnológico de Canarias, Las Palmas, Spain; 5 Institute of Natural Products and Agrobiology (IPNA-CSIC), Av. Astrofisico Francisco Sánchez, San Cristóbal de La Laguna, Santa Cruz de Tenerife, Spain; National Autonomous University of Mexico Institute of Geophysics: Universidad Nacional Autonoma de Mexico Instituto de Geofisica, MEXICO

## Abstract

The 2021 Tajogaite eruption on La Palma devastated banana production, a key crop, with a 50% loss (53,000 tons). Concerned about potential contamination from volcanic ash and magma, we investigated the elemental composition of bananas from the eruption area and control sites. Inductively Coupled Plasma Mass Spectrometry (ICP-MS) analysis quantified both essential and non-essential mineral elements, including potentially toxic elements identified by the Agency for Toxic Substances and Disease Registry (ATSDR), as well as rare earth elements (REEs) and other trace elements that are scarcely studied under volcanic conditions. This approach allowed for spatial and temporal comparisons. Results showed a decrease in element levels post-eruption; however, samples from the volcanic area still exhibited elevated concentrations of Fe, Co, Cd, Al, Ba, Ni, Sn, Sr, Ti, V, and REEs. Control samples from unaffected islands with higher anthropogenic pressure showed elevated levels of Mn and Mo. Despite the increased element levels, banana consumption remains safe and constitutes a valuable source for the recommended daily intake of Mo and Co. Most toxic elements were present at less than 1% of the tolerable daily intake (TDI), with the highest values for As and V reaching 3%, and no risk was associated according to the margin of exposure approach. This eruption highlights the need for continuous monitoring in volcanic regions to safeguard public health and food safety.

## 1. Introduction

Volcanic eruptions, often catastrophic, drastically modify environments. Beyond physical changes pyroclastic flows, high‐speed, high‐temperature currents of gas and tephra, deposit fine abrasive particles and volatiles directly onto the landscape, leading to the near-instantaneous release of trace metals and metalloids (e.g., As, Se, Pb) into soils and water bodies, and causing sharp spikes in potentially toxic elements in groundwater and sediments [1,2]. By contrast, lava flows, due to their insulating and burying effects on the soil, release soluble elements such as Ca^2^ ⁺ , Mg^2^ ⁺ , Ni, or V more gradually over months to years, primarily through weathering processes including hydrolysis and oxidation under environmental conditions. These events negatively impact agriculture, health, and food safety, with potential long-term consequences [3–6]. Therefore, volcanic ash, enriched with toxic elements (e.g., arsenic, cadmium, mercury ornickel), poses risks to biota and alters crop nutrition [6–8].

On September 19, 2021, the Tajogaite volcano erupted in La Palma Island (Canary Islands, Spain), and remained active for 85 days. La Palma is the westernmost island of the Canary Archipelago, a group of islands situated in the Atlantic Ocean, facing the coasts of Morocco and Western Sahara. The Canary Islands have a volcanic origin, with the easternmost islands being the oldest geologically, and those in the west still forming due to ongoing volcanic activity [9]. The island of La Palma has an economy based on the primary sector, with a notable focus on banana cultivation. In fact, 32% of the banana production in the Canary Islands comes from the island of La Palma [10]. As a result, a substantial portion of the bananas exported to European markets from the Canary Islands archipelago are cultivated on that island. The volcanic eruption significantly impacted banana production, not only because farmlands were destroyed by lava, but also due to potential toxicological consequences from the volcanic material on locally grown foodstuffs.

Bananas, rich in potassium, vitamin C, and magnesium, are influenced by environmental factors,particularly volcanic ash deposition, which transiently shifts soil pH, delivers pulse inputs of base cations (e.g., K ⁺ , PO₄^3^ ⁻ , SiO₂), and mediates competitive adsorption that can immobilize essential elements (e.g., Mg^2^⁺ or Ca^2^⁺) [11]. However, it may also lead to the accumulation of toxic element, affecting fruit quality and posing health risks with excessive consumption [12]. Besides, recent studies have identified certain elements, previously not classified as harmful, specifically rare earth elements (REEs) such as lanthanum, gadolinium, and cerium, along with other microelements (MEs), as emerging environmental pollutants. These elements, of low abundance in the Earth’s crust, are increasingly released into the environment during volcanic eruptions and are accumulating in global populations, further driven by their extensive use in the high-technology industry [13]. Potential adverse effects on ecosystems and human health, including neurotoxicity and genotoxicity, are currently under investigation [14]. While the banana peel is known for its protective properties, it also exhibits a remarkable ability to absorb a range of substances, including mineral elements [15]. Consequently, bananas exposed to volcanic ash may accumulate airborne elements not only through surface adsorption, but also via cuticular partitioning within the cutin–wax matrix and diffusion through microcracks in the epicuticular wax layer. Vascular bundles, consisting of xylem and phloem conduits, may facilitate the translocation of absorbed ions from the peel to the pulp during fruit development and storage. This process can enhance the transfer of magmatic-origin elements into the edible portion of the banana, depending on the degree of weathering and the mineralogical composition of the soil [16,17].

Previous assessments revealed that, prior to washing (a common practice before commercialization), bananas contained elevated levels of various magmatic elements. The washing process resulted in a significant reduction in the concentration of these elements, such as cadmium, lead and nickel, rendering the bananas safe for consumption [18]. Nutritional analysis of the washed bananas revealed higher levels of essential elements for human health, such as molybdenum and cobalt, in fruits exposed to the volcanic eruption compared to pre-eruption samples, resulting in a beneficially higher contribution to the recommended intake of these micronutrients [12]. However, the previous study focused on bananas sampled at the start of the eruptive period, cultivated before that time, leaving open questions about the long-term effects of volcanic materials on banana plants and fruit quality. Continuous monitoring of banana nutritional and toxic element composition in volcanic regions is essential to assess human health risks, develop safety recommendations, and understand accurately the consequences of volcanic eruptions on agriculture and health [5].

To address the uncertainties highlighted in the previous study, a comparative analysis was conducted on the nutritional, toxic, rare earth, and other scarcely studied elements in foodstuff cultivars grown in a volcanic environment. Banana samples were collected from both affected and unaffected areas during the eruption, at its conclusion, and in the post-eruption period to evaluate the influence of volcanic emissions on their mineralogical profile. Elemental analysis was performed using Inductively Coupled Plasma Mass Spectrometry (ICP-MS), enabling the assessment of dietary intake under different exposure scenarios.

## 2. Materials and methods

### 2.1. Sampling

Control samples were collected from farms unaffected by the volcanic eruption. These control samples were obtained from other islands (n = 57): Tenerife (n = 38), Gran Canaria (n = 12), El Hierro (n = 5), and La Gomera (n = 2).

For samples affected by volcanic emissions, the sampling design was opportunistic random, constrained by the availability of official permits issued by the availability of official issued by the relevant authorities for each area. Only safely accessible farms were included in the study, with areas at risk of landslides, ground collapse, or residual pyroclastic activity were deliberately excluded. Thus, bananas exposed to the volcanic emissions were collected by authorized personnel and transported to multiple processing plants. These facilities received bananas from 8 out of the 14 municipalities on the island (n = 63). The distance from the farms to the volcano was measured, resulting in the categorization of farms into two distinct groups: those located 5–10 km from the volcano (n = 25) and those positioned 20–25 km away (n = 38). The sampling was conducted at 3 different time points: a) on day 40 of the volcanic eruption, nearly at the midpoint of the eruption’s course (12 samples from affected and 12 from unaffected areas); b) on day 92, one week after the conclusion of the eruption was officially declared (19 samples from affected and 26 from unaffected areas); and c) between 8 and 12 months after the end of the volcanic eruption (32 samples from affected and 19 from unaffected areas). As a result, the bananas were categorized into four groups: “Controls”, “During Eruption”, “End of Eruption”, and “After Eruption”For the samples collected during and immediately after the volcanic eruption, whole banana bunches were cleaned on-farm using pressurized air to remove as much volcanic ash as possible.

Upon arrival at the processing plant, the banana bunches were first submerged in specially prepared water tanks equipped with high-pressure jets to mechanically remove any surface-adhering volcanic ash. Once de-stemmed, the banana hands underwent the standard post-harvest washing process (consistently applied to both post-eruption and control samples) on a conveyor belt, performed prior to packaging and transport.. This included the application of fungicides, such as azoxystrobin or thiabendazole, aimed at preventing crown rot and ensuring the quality and longevity of the fruit. Thus, the bananas gathered were in their market-ready condition. The control samples adhered to the established self-control protocol for pesticide residues before export, in accordance with the customary procedures of the Department of Environmental Analysis at the Instituto Tecnológico de Canarias. Each sample consisted of two clusters of bananas, selected from different positions within the bunch: one from the top and one from the bottom. The combined weight of each sample was approximately 2 kg. This sampling strategy was designed to capture intra-bunch variability and ensure that the measured elemental concentrations accurately reflect typical consumer exposure. On the day of collection, the samples were transported to the laboratory and prepared for analysis the following day. The whole bananas (comprising both peel and flesh) and the pulp alone were processed separately for independent sample analysis.

### 2.2. Standards and elements

The concentration levels of 50 elements in banana homogenates were determined using pure elemental standards in acid solution (5% HNO₃, 100 mg/L; CPA Chem, Stara Zagora, Bulgaria). The analysis included a broad spectrum of elements, categorized as follows: 1) Essential mineral elements have been considered based on their essential physiological functions for human health: cobalt (Co), copper (Cu), iron (Fe), manganese (Mn), molybdenum (Mo), selenium (Se), and zinc (Zn); 2) Elements with significant toxicity and potential exposure risks have been selected based on the 2023 ATSDR Substance Priority List, which considers substances that pose the most significant potential threat to human health based on their frequency, toxicity, and potential for human exposure [19], divided into highly toxic elements: arsenic (As), cadmium (Cd), mercury (Hg), and lead (Pb), and potentially toxic elements: silver (Ag), aluminum (Al), barium (Ba), beryllium (Be), chromium (Cr), nickel (Ni), palladium (Pd), antimony (Sb), strontium (Sr), thorium (Th), thallium (Tl), uranium (U), and vanadium (V); 3) Rare earth elements (REEs) and other microelements (MEs) were selected based on their technical classification as rare earth elements and other trace elements of low abundance in the Earth’s crust [20]. These elements are valued and considered as emerging contaminants for their common uses in industrial, technological, medical, and zootechnical applications, and were included in this case because they are scarcely studied in the context of volcanic episodes:: gold (Au), bismuth (Bi), cerium (Ce), dysprosium (Dy), erbium (Er), europium (Eu), gallium (Ga), gadolinium (Gd), holmium (Ho), indium (In), lanthanum (La), lutetium (Lu), niobium (Nb), neodymium (Nd), osmium (Os), praseodymium (Pr), platinum (Pt), ruthenium (Ru), samarium (Sm), tin (Sn), tantalum (Ta), terbium (Tb), titanium (Ti), thulium (Tm), yttrium (Y), and ytterbium (Yb). Tl has been considered as an element with recognized toxic potential due to its ability to induce DNA damage and cause genetic and epigenetic alterations [21]. Finally, it is important to highlight that five of the selected essential elements (Co, Cu, Mn, Se, and Zn) are listed on the 2023 ATSDR Substance Priority List. This underscores that, although these elements play vital physiological roles and are regulated by the body, excessive exposure can still pose potential health risks, emphasizing the need to maintain an appropriate mineral balance.

### 2.3. Analytical procedure

The analytical procedure involved homogenizing the flesh of six bananas from each of the two clusters that constituted each sample, using a household food processor equipped with ceramic blades that were thoroughly cleaned with nitric acid to prevent cross-contamination. Additionally, six bananas from each cluster were processed without separating the skin, aiming to examine the total element content within the fruit. Samples were freeze-dried, stored in polypropylene plastic containers for analysis, and transported to the Toxicology Unit of the University of Las Palmas de Gran Canaria, where multielement analysis was performed.

Duplicate digestions of each subsample were performed as follows: one gram of the homogenized sample underwent acid digestion using 3mL of sub-boiled distilled 65% nitric acid and 7mL of ultra-pure water sourced from a Milli-Q system. The digestion process for mineralization was conducted in a microwave digester (Ethos Up, Milestone SRL, Italy). The microwave system was set up to operate at 1800 W in four different steps, reaching a maximum temperature of 180°C before cooling and a total operation time of 100 min,as described in detail in our previous research [18]. The digested samples were transferred to metal-free tubes, and three aliquots from each subsample were prepared for ICP-MS analysis. These aliquots were placed in autosampler vials and diluted to a final concentration of 4% (v/v) nitric acid in a total volume of 3 mL. Consequently, six independent measurements were conducted for each sample to determine the median value for each element.

Instrumental analyses were conducted using an Agilent 7900 ICP-MS (Agilent Technologies, Tokyo, Japan), equipped with standard nickel cones and a MicroMist glass concentric nebulizer. The 4th generation Octopole Reaction System (ORS4) operated in helium (He) mode for all elements to minimize interferences from low-mass elements, enhancing instrumental detection limits. To avoid isobaric interferences, isotopes with distinctly different mass values were selected, ensuring that the most abundant isotope of one element does not interfere with the measurement of another. Each day before operation, the instrument was tuned using a solution containing cesium, cobalt, lithium, magnesium, thallium, and yttrium (Agilent Technologies, Palo Alto, CA, USA). This tuning step optimized instrument performance by confirming sensitivity for each isotope (in counts per second), assessing relative standard deviation (RSD) values, and ensuring that oxide and doubly charged ion ratios met recommended specifications. Two separate standard curves (0–300 ng/mL range) were prepared to minimize interferences from doubly charged ions of rare earth elements and ensure maximum accuracy: (a) one for essential and toxic trace elements, including elements stabilized in hydrochloric acid (Os, Pt, Au, Pd, and Ru); and (b) another for REEs and remaining microelements. To prevent memory effects, a cleaning solution of 2.0% HNO₃ and 1% HCl was used between samples. Quantification was performed using MassHunter v.4.2 ICP-MS Data Analysis software (Agilent Technologies).

An internal standard solution comprising scandium (Sc), germanium (Ge), rhodium (Rh), and iridium (Ir) was added to each vial prior to sample analysis, calibration, and quality control to achieving a final concentration of 40 ng/mL. This practice is standard in ICP-MS for monitoring counts per second (CPS) reproducibility, RSD values, and isotope recovery rates. These elements are commonly used as internal standards due to their low concentrations in most sample matrices and their effectiveness in correcting for matrix effects and instrumental drift. Germanium (Ge) was excluded from the REEs calibration curve after each sequence to avoid interference from doubly charged neodymium (Nd^2^⁺) and samarium (Sm^2^⁺) ions (m/z 71–75), thereby improving measurement accuracy. The entire procedure underwent in-house validation before its application in the samples analyses. Quality parameters, including linearity (correlation coefficient, R^2^), limits of detection and quantification, precision, and accuracy (coefficient of variance, CV%), were assessed as previously described [18]. Instrumental Limits of Detection (LODs) and Limits of Quantification (LOQs) were calculated as the concentration of the element that produced a signal that was three and ten times higher than the average of the blanks, respectively [22]. Sample LOQs were calculated by multiplying the instrumental LOQ by the dilution factor suffered by the sample during the digestion procedure. The details of the LOD and LOQ are provided in S1 Table. As digestion was completed with no remaining organic matter from banana samples and no certified reference material (CRM) is commercially available for this matrix, the accuracy and precision of the proposed methodology were assessed through recovery studies using an acid solution (4% HNO₃) fortified with all the elements studied at three different concentration levels (0.5, 5, and 50 ppb). Calibration curves generally displayed excellent regression coefficients (≥0.995), and recoveries values ranged from 82 to 117% for all the elements. Precision was generally below 5% at the medium and highest concentration levels. However, for certain specific measurements of some elements (Cu, Ni, Ga, Sn, Ta), precision reached 8–9% at the lowest concentration levels. These measurements were ultimately excluded from the analysis, as the accepted values were based on RSD lower than 5%, in accordance with recommendations and the performance of our ICP system.

In addition, digested blanks and reagent blanks (prepared using the same acid solution as that used for standards, calibration, and samples) were included as quality controls in each batch to identify any contamination from reagents or digestion vessels. Furthermore, to ensure analytical quality the three tailored matrices were incorporated to evaluate recovery and accuracy. Analyte concentrations were considered acceptable when recoveries (%R) ranged between 80–120% and RSD values remained below 5%, in accordance with criteria previously validated in the scientific literature [23]. As six independent measurements were prepared per sample, with three automatic readings per vial, outlier values could be excluded, and the median value was used to express the element concentration. S2 Table contains the unprocessed/raw data resulting from the analytical procedures.

### 2.4. Estimation of element intake and risk-benefit analysis

To estimate element intake, banana consumption data from the European population, the Spanish population [24], and the Canary Islands population, where banana consumption is the highest in Europe [25] were considered. Element median concentrations (µg/kg) found in the banana’s edible portion were multiplied by daily consumption levels at the 50^th^ and 97.5^th^ percentiles (grams per day) and normalized by the average body weight (kg) of the population to calculate the Estimated Daily Intake (EDI). The resulting EDI) values obtained for essential elements for each consumption scenario (average and high consumers) were compared to the population reference intake (PRI) values set by the European Food Safety Authority (EFSA) as dietary reference standards [26–28]. The PRI represents the daily dietary intake level of a nutrient considered adequate to fulfill the requirements of 97.5% of healthy individuals within specific life stages and sex groups. In cases where EFSA has not specified a Recommended Dietary Allowance (RDA), the Adequate Intake (AI) was used as reference value. Therefore, for each element considered essential for the maintenance of human health, we have estimated the percentage of the RDA or AI values reached, expressed as the Recommended Daily Intake (RDI). Moreover, for essential elements where the Estimated Daily Intake (EDI) exceeded the RDI, the Tolerable Upper Intake Level (UL) was also considered. The UL represents the maximum chronic daily intake of a nutrient from all sources that is unlikely to pose a risk of adverse health effects in humans [29].

For the estimation of the risk posed by highly and potentially toxic elements, the Toxic Reference Values (TRVs) applied in this study were based on the U.S. Environmental Protection Agency’s (EPA) non-carcinogenic tolerable daily intake (TDI) values [30] and their integration with EDI, with the exception of cadmium (0.36 µg/kg body weight/day for renal effects), which was established by EFSA [31]. Nevertheless, Pd and Th were excluded from the risk analysis, as no established TRVs are available for these elements. Moreover, to estimate potential health risks, including carcinogenic and genotoxic effects, the Margin of Exposure (MOE) was calculated using the Benchmark Dose Lower Confidence Limit (BMDL), which is the lower confidence limit of a point on the dose-response curve associated with adverse effects. This was integrated with calculated EDI (MOE = BMDL/EDI). The lowest BMDL values (expressed in μg/Kg b.w/day) were selected in accordance with the precautionary principle for three elements: inorganic arsenic, which have been recently updated (0.06), covering risks for skin cancer as well as lung and bladder cancers, skin lesions, ischemic heart disease, chronic kidney disease, respiratory disease, reproductive issues, infant mortality, and neurodevelopmental effects; nickel (4.3) for systemic contact dermatitis; and lead (0.50) for developmental neurotoxicity in children, and 0.63/1.50 for nephrotoxicity and cardiovascular effects in adults, respectively [32–34]. For this purpose, high consumers are considered, and an inorganic arsenic (i-As) content of 70% relative to the total has been assumed, based on the results collected for the fruit category [35].

REEs and other MEs considered in this study lack official TDI values. Nevertheless, certain studies suggest an acceptable daily intake of 70 µg/kg body weight/day for rare earth oxides [36].. Consequently, these elements have been collectively assessed in the risk analysis, as previously done [37], which are the following: Ce, Dy, Er, Eu, Gd, La, Lu, Nd, Pr, Sm, Tb, Tm, Y and Yb. Another report conducted in 2022 for the risk assessment of tea established a reference value of 51.3 μg/kg body weight per day for the sum of all rare earth elements [38]. Both values have been considered as TDIs in the risk assessment.

### 2.5. Statistical analysis

Descriptive analyses were conducted for all variables. Medians and ranges were calculated for continuous variables. Proportions were calculated for categorical variables. Normal distribution of data was evaluated with the Kolmogorov-Smirnov test. Due to the non-normal distribution of data and the presence of undetected values, particularly among REEs and other MEs, non-parametric tests were employed. Kruskal-Wallis and Mann-Whitney U tests were used for overall and pairwise comparisons, respectively. Differences in the categorical variables were tested by the Chi-square test. Data below the LOQs but above the LODs were assigned a random value between these two limits. This approach preserves data variability and minimizes bias that may result from substituting values below the LOQ with zeros, the LOD, or fixed estimates. By doing so, it enhances the accuracy of descriptive statistics and supports more robust and reliable statistical inferences [18]. Data below the LODs were considered undetected. Statistical analyses were conducted using GraphPad Prism v9.3 software (GraphPad Software, CA, USA). Significance was established as p ≤ 0.05.

## 3. Results and discussion

When evaluating the proximity of plantations to the volcanic eruption site, an increase in the concentration of elements was observed as the distance from the volcano decreased. Thus, arsenic levels were significantly higher in closer areas than in more distant areas (2.07 and 0.59 ng/g; P value = 0.038). Volcanic materials readily absorb harmful metals and metalloids [39], potentially facilitating their transfer to adjacent banana plants, aligning with our findings. In this case, the observed pattern is likely due to the deposition of ash and pyroclastic material enriched in amorphous Fe, Mn, or Al oxides, which, under fluctuating redox conditions and gas–particle interactions, can alternately immobilize or release metalloids [40]. This dynamic may help explain the modulation of As⁵⁺ and As^3^ ⁺ species and their bioavailability to banana roots near the volcano. In addition, higher concentrations of sum of the REEs were found in bananas from plantations closer to the volcano compared to those farther away (44.67 and 5.24 ng/g, respectively), although these differences were not statistically significant. This pattern may be attributed to enhanced adsorption–desorption dynamics in young volcanic soils, which facilitate the release of REEs into nearby crops. Plantations in closer proximity to the eruption were directly impacted by the deposition of volcanic ash and pyroclastic material, which are often enriched in light rare earth elements (LREEs) such as La, Ce, Nd, Eu, and Gd. These elements tend to be more abundant due to their lower atomic masses, higher solubility, and greater mobility under alkaline conditions, becoming concentrated in volcanic ash through hydrothermal alteration and weathering processes [41]. Soil pH and the presence of Mn-Fe-oxyhydroxides also play significant roles in REE mobility and accumulation [42]. Plant species in similar environments often accumulate more REEs in roots than in shoots or leaves, due to selective uptake and internal fractionation processes [43]. Additionally, translocation and bioaccumulation factors vary between plant species and are influenced by the physicochemical characteristics of the substrate [43].

No significant differences were observed among control samples from different territories across sampling periods (n = 57). Thus, bananas from other islands were treated as a homogeneous group.

### 3.1. Effect of the volcanic eruption on the concentration and occurrence of essential elements. Dietary intake and nutritional assessment

The concentrations of the essential elements considered in this study, both in the whole banana and in the pulp, as well as the variations relative to the different time points in relation to the dynamics of the volcanic eruption, are presented in Table 1. Iron and zinc showed the highest concentrations. Manganese was more concentrated in the whole fruit than copper, indicating greater Mn presence in the peel, which was less prominent in the case of Cu. This peel enrichment of Mn aligns to reports indicating that banana peel tissues sequester excess Mn via vacuolar storage and epidermal oxidation defense, particularly under acidified, ash‐rich soils typical of volcanic region [44]. Molybdenum was higher in the pulp concentration, consistent with our prior study [18]. Selenium and cobalt had lower concentrations, below 10 ng/g in both the whole fruit and in the pulp, suggesting a limited source of these elements in the banana. Se was also the only essential element not detected in all the samples studied (Table 1). Prior research in Africa on *Musa spp*., the genus to which the banana plant belongs, reported similarly high concentrations of essential element in the peel, including calcium, potassium and sodium, as well as micronutrients like zinc and manganese, which were 3–8 times higher in peels, respectively [45]. Mn and Cu concentrations have been reported to be higher in the pulp of bananas and plantains, in line with our findings [45]. The present results showed higher Cu and Zn levels, and similar Mn concentrations compared to *Musa spp.* bananas from Nigeria (mean values of 160, 130, and 930 ng/g, respectively). However, Se and Mn were lower than those reported values in Thai bananas [46]. Pulp samples from the control group samples showed manganese and iron concentrations comparable to those of bananas grown in the Canary Islands and Ecuador, under both greenhouse and outdoor cultivation, and across conventional and organic production systems in Tenerife [47]. These results are linked to the fact that banana cultivation is strongly influenced by soil properties, environmental conditions and human activities such as industrialization, urbanization, and the use of chemical inputs. These factors can potentially lead to contamination, affecting both the quality and safety of the crop [46,48].

**Table 1:** Comparison of essential element levels in whole bananas and banana pulp during and after a volcanic eruption. Data are expressed in ng/g fruit.

WHOLE BANANA																	
	During eruption (n = 12)				At the end of eruption (n = 19)				After eruption (8–12 months) (n = 32)				Control group (n = 57)				Statistical significance
Element	Median	Upper limit	Lower limit	>LOD (%)	Median	Upper limit	Lower limit	>LOD (%)	Median	Upper limit	Lower limit	>LOD (%)	Median	Upper limit	Lower limit	>LOD (%)	*P*^*a*^/*P*^*B*^
Fe	5141.8	25714.6	2971.4	100	6880.2	12999.3	5421.5	100	3229.7	3704.7	2913.5	100	2624.9	3096.7	2121.6	100	0.002/n.s.
Zn	1057.7	1309.6	620.9	100	1209.1	1579.8	987.9	100	1073.4	1355.3	801.4	100	1171.5	1442.1	972.1	100	n.s./n.s
Cu	431.1	490.3	363.4	100	498.1	574.1	388.5	100	416.4	493.5	339.9	100	462.4	547.3	397.0	100	n.s./n.s
Se	4.7	16.6	2.2	83.3	6.4	7.2	4.1	100	0.0	2.4	0.0	60.6	0.3	5.2	0.0	67.4	n.s./n.s
Mn	822.8	1204.0	619.6	100	896.5	1233.7	621.8	100	494.2	672.9	403.6	100	876.6	1192.5	590.9	100	<0.000/n.s
Mo	86.3	98.6	73.5	100	76.9	96.1	54.9	100	45.4	73.5	16.5	90.9	96.4	140.4	78.2	100	<0.000/n.s
Co	2.9	15.1	1.2	100	3.8	9.0	2.1	100	1.8	2.3	1.2	100	1.3	1.7	0.8	100	0.043/n.s
**BANANA PULP**																	
	**Median**	**Upper limit**	**Lower limit**	**>LOD** **(%)**	**Median**	**Upper limit**	**Lower limit**	**>LOD** **(%)**	**Median**	**Upper limit**	**Lower limit**	**>LOD** **(%)**	**Median**	**Upper limit**	**Lower limit**	**>LOD** **(%)**	***P*** ^***a***^ ***/P*** ^***B***^
Fe	3166.5	4202.6	2822.1	100	5042.9	5469.8	3903.7	100	3631.9	4361.7	3321.3	100	2680.5	4010.4	2487.8	100	0.037/n.s
Zn	1216.2	1428.2	798.2	100	1399.9	1890.6	1160.9	100	1394.6	1570.2	1002.0	100	1176.2	1255.9	1048.9	100	n.s./n.s
Cu	591.7	664.9	469.4	100	691.2	769.8	517.7	100	621.0	704.0	510.9	100	683.5	716.4	564.4	100	n.s./n.s
Se	2.6	4.0	0.4	83.3	3.8	7.1	0.3	75	0.0	1.9	0.0	57.6	0.2	3.0	0.2	69.6	n.s./n.s
Mn	504.5	598.3	436.1	100	614.1	896.3	465.1	100	414.5	523.9	364.6	100	614.9	706.7	545.7	100	<0.000/n.s
Mo	111.3	143.2	87.1	100	106.0	133.0	73.5	100	71.9	100.4	32.1	96.9	133.9	191.7	119.9	100	<0.000/n.s
Co	1.7	2.6	1.1	100	2.0	2.8	1.6	100	1.8	2.9	1.0	100	1.4	2.1	0.9	100	0.042/n.s

^a^*P value* resulting from the comparison between the median values of bananas after eruption and control group (Mann-Whitney test).

^b^*P value* resulting from the comparison between the detection percentage (>LOD) of bananas after eruption and control group (Chi-square test).

n.s.: not significant.

To hell elucidate the presents results, it is important to note that soil pH, organic matter content, and nutrient availability are critical factors regulating the uptake and distribution of essential elements in banana plants. While the optimal pH range for nutrient absorption is 6.8–7.2, more acidic soils (pH < 5.5) may increase the solubility of micronutrients such as Mn and Fe but can negatively impact plant development if not properly managed [49]. Elevated organic matter enhances nutrient bioavailability and crop yields by promoting microbial activity, accelerating litter decomposition, and improving nutrient cycling, thereby supporting plant growth. However, in agricultural volcanic soils, trace metal contamination generally reduces soil enzyme activities and microbial biomass [50], though it may also create conditions that stimulate certain enzyme activities and foster resilient, beneficial microbial communities.

**Fig 1:**
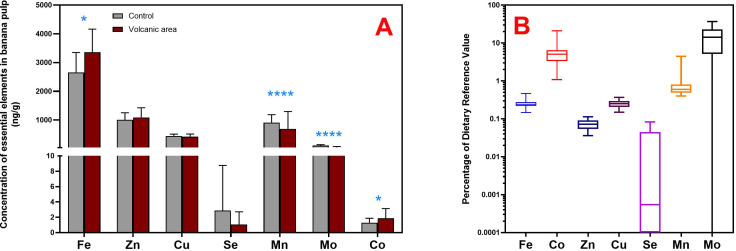
(A) Comparison of the essential elements content in banana pulp homogenates from samples collected 8–12 months after the volcanic eruption and from the control group. *P < 0.05; ****P < 0.001. (B) Box plots showing the percentage of the Dietary Reference Value (Recommended Daily Intake) of essential elements through the consumption of bananas from La Palma Island grown after the eruption period and influenced by volcanic emissions (8-12 months). The lines connect the medians, the boxes cover the 25th to 75th percentiles, and the minimal and maximal values are shown by the ends of the bars.

Considering the volcanic impact on banana composition, zinc, copper, and selenium levels in both whole fruit and pulp showed no differences between eruption and samples collected 8–12 months later (Fig 1A). Concentrations of Fe, Mn, and Mo significantly increased in whole bananas and pulp from the eruption period till later stages. Notably, Fe levels in whole fruit doubled during this time (Table 1), highlighting the impact of soil composition and agro-climatic conditions on mineral content. This finding suggests that Fe levels in bananas appear to be more influenced by external factors (such as soil conditions, the environment, or other factors related to the volcanic eruption) than by the specific banana cultivar grown [47]. In this context, the integration of volcanic material can alter the distribution coefficients (Kd) of essential elements in soils by modifying key edaphic parameters, including the abundance and crystallinity of iron and manganese oxides, and introducing alkaline earth cations (e.g., Ca^2^⁺ and Mg^2^⁺), that may precipitate or compete with trace metals for sorption sites [51,52].

Banana pulp from the post-eruptive area exhibited elevated levels of iron (Fe) and cobalt (Co) compared to the control group (Fig 1A), likely as a result of the rapid weathering of volcanic ash, which releases soluble Fe^2^⁺ and Co^2^ ⁺ ions. Subtropical rainfall and acidic soil conditions (pH < 6) further enhance their bioavailability and plant uptake. Conversely, pulp from the control group exhibited significantly higher concentrations of manganese and molybdenum. Notably, Mo isotope fractionation in soils is influenced by redox conditions, organic matter content, and atmospheric inputs, which may serve as indicators of climate-driven changes in the weathering environment [53]. Increased Mn concentrations may be linked to factors such as groundwater irrigation and the use of fungicides, particularly those combating *Fusarium spp* [54]. A study conducted in a tropical banana production area in Mexico identified high manganese levels in soils, surface water, and groundwater, likely linked to dithiocarbamate fungicides like Mancozeb, which degrade into manganese-containing byproducts [54].. In the Canary Islands, such fungicides are not currently in use, according to local agricultural records and expert consultations. Instead, Mn dynamics are more likely influenced by practices such as micronutrient fertilization, irrigation with mineral-rich water, and the application of organic amendments. As mentioned, edaphoclimatic factors affect soil pH, redox conditions, and Mn bioavailability [55].

Regarding the dietary intake assessment, despite their low molybdenum and cobalt content, bananas can still contribute significantly to the recommended daily intake (RDI) when typical consumption is considered (Fig 1B). Consistent with previous findings [18],it is nutritionally noteworthy that high consumers of post-eruption bananas can achieve up to 37% and 21% of the recommended daily intake (RDI) for Mo and Co, respectively. Nevertheless, manganese intake does not reach 10%, even at the highest consumption percentile, and bananas contribute minimally to the adequate intake of iron, zinc, copper, and selenium, providing less than 1% of the RDI for average consumers, even after prolonged exposure to volcanic material. Moreover, all essential elements analyzed remained well below the established upper limits, suggesting no adverse health risks through their intake. It is important to note, however, that statistical significance does not necessarily translate into biological or nutritional relevance. This underscores the need for further research into the potential health implications of variations in mineral content, particularly when considering the wide interindividual variability in mineral absorption. Factors such as age, nutritional status, biochemical profile, and the presence of specific deficiencies can all affect the efficiency of mineral uptake and metabolism.

### 3.2. Effect of the volcanic eruption on the concentration and occurrence of highly/potentially Dietary intake and risk assessment

#### 3.2.1. Highly toxic elements.

**Fig 2:**
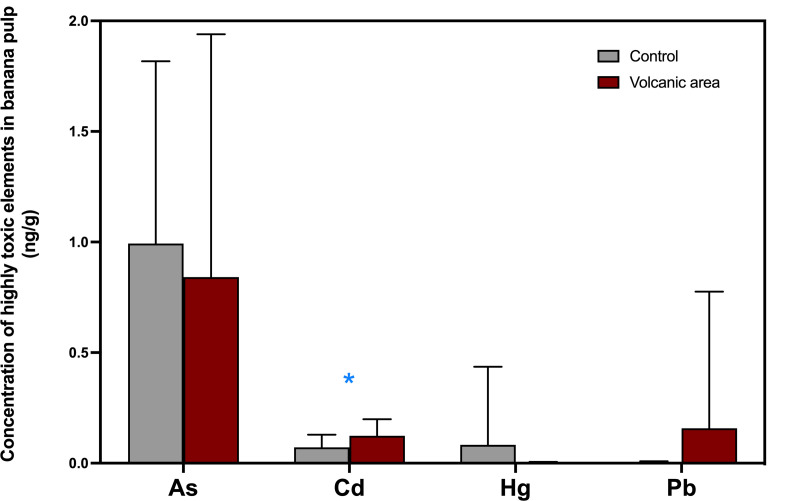
Comparison of the highly toxic elements content in banana pulp homogenates from samples collected 8–12 months after the volcanic eruption and from the control group. *P < 0.05.

In general, a decrease in the presence of these elements was observed among the samples collected during and after the eruption, except for Cd. Mercury was the least frequently detected element, particularly in the pulp samples (Table 2). Arsenic had the highest concentration, nearly 10 times greater than those of cadmium (median = 0.9 and 0.1 ng/g pulp) in post-eruption samples. While bananas generally show limited arsenic translocation to fruit, uptake can occur via phosphate transporters under certain environmental conditions [56]. Pb showed the highest concentration in fruits exposed to the volcano during its activity, both in whole banana and in the pulp (1.2 and 0.8 ng/g, respectively; Table 2). However, Pb was more frequently detected in non-exposed samples than in those collected months after the eruption, possibly due to greater industrialization on these islands. Only Cd showed a significant difference in concentration, reaching its highest level at the end of the eruption, but only in the pulp (P = 0.018), as shown in Fig 2 No other significant differences were observed. These highly toxic elements are studied individually, as they are also regulated by food legislation. The detected levels of cadmium and lead in fruits, including bananas, were within legal limits (0.050 and 0.10 mg/kg, respectively) [57,58], and were lower than those reported in samples from developing countries [12,59–61]. However, it is important to emphasize that regulatory compliance does not necessarily equate to the absence of health risks. This is particularly relevant in cases of chronic exposure and among vulnerable groups such as children and pregnant women, who exhibit greater physiological sensitivity and higher absorption rates. Volcanic eruptions release highly toxic elements [62,63]. In that sense, copper, cadmium, chromium, mercury, and lead have been detected in soil and ash leachates in volcanic eruptions in Mexico [4]. However, our results showed low concentrations of these elements which may be attributed to the removal efficiency of banana peels. The peel acts as an effective barrier, limiting the transfer of toxic metals to the pulp through a combination of chemical complexation (via pectin and cellulose), precipitation of metals as insoluble compounds such as carbonates or phosphates, and the presence of a lignified structural layer [64,65]. This multi-layered defense reduces the effective surface area for metal uptake in tissues, even under exposure to volcanic inputs. Accordingly, the use of banana peels as biochar has been well documented, owing to their high adsorptive capacity and favorable biosorption properties [66]. The bananas studied contained low levels of these elements, posing no health risk. Since the maximum total daily intake (TDI) value for high consumers is primarily represented by arsenic (accounting for 3% of the established toxic level; data not shown), bananas would be considered a safe dietary choice. Our findings suggest that the enrichment of soil organic matter with toxic elements such As, Cd, or Pb from volcanic eruptions, as observed in calcium carbide-enriched banana varieties [67], does not pose an undue concern. Meta-analyses on fresh and processed fruits generally confirm that element ingestion in these foods does not pose a risk to the population, as assessed by the target hazard quotient (THQ) [46,59,60,68]. However, in certain studies carried out in Ecuador, South Africa, and Pakistan, lead levels exceeded the recommended THQ, with sources linked to smelting activities, vehicle emissions, and open dumping of municipal waste. These findings raise concerns about health issues related to Pb exposure, such as neurodevelopmental and cardiovascular disorders [12,61,69]_._ Regarding the MOE estimation, our values of 50,000, 63,000, and 150,000 for Pb indicate a high margin of safety (>10,000) in relation to adverse effects on the nervous, renal, and cardiovascular systems, respectively [34]. Inorganic arsenic intake in banana samples from the volcano is 3% of the BMDL proposed for increased skin, lung, and bladder cancer, among other effects. The MOE value for i-As was 30.7, indicating a higher safety level compared to data reported for the European population (0.2–0.9). However, no MOE of low concern has been established based on human cancer data [32].

**Table 2:** Analysis of highly toxic element concentrations in whole bananas and banana pulp during and post-volcanic eruption. Data are expressed in ng/g fruit.

WHOLE BANANA																	
During eruption (n = 12)	At the end of eruption (n = 19)	After eruption (8–12 months) (n = 32)	Control group (n = 57)	Statistical significance
Element	Median	Upper limit	Lower limit	>LOD (%)	Median	Upper limit	Lower limit	>LOD (%)	Median	Upper limit	Lower limit	>LOD (%)	Median	Upper limit	Lower limit	>LOD (%)	*P*^*a*^/*P*^*B*^
As	1.0	2.6	0.3	100	1.1	2.3	0.6	85	0.6	0.9	0.0	72.7	0.7	1.6	0.4	86.9	n.s./n.s.
Cd	0.1	0.3	0.1	100	0.2	0.4	0.1	100	0.1	0.2	0.0	72.7	0.1	0.1	0.0	80.4	n.s./n.s.
Hg	0.3	0.4	0.0	58.3	0.0	0.0	0.0	15	0.0	5.7	0.0	33.3	0.0	0.0	0.0	23.9	n.s./n.s.
Pb	1.2	3.3	0.7	100	0.0	3.1	0.0	40	0.0	0.0	0.0	15.1	0.0	0.0	0.0	43.5	n.s./0.013
**BANANA PULP**																	
n.s./n.s.	As	0.5	1.3	0.2	100	0.8	2.3	0.4	80	0.6	1.0	0.1	75.8	0.9	1.7	0.3	80.4
0.018/n.s.	Cd	0.1	0.2	0.1	100	0.2	0.3	0.1	100	0.1	0.2	0.1	87.9	0.1	0.1	0.0	82.6
n.s./n.s.	Hg	0.0	0.4	0.0	33.3	0.0	0.0	0.0	15	0.0	0.0	0.0	6.1	0.0	0.0	0.0	8.7
n.s./0.014	Pb	0.8	1.0	0.5	100	0.0	0.0	0.0	25	0.0	0.0	0.0	15.2	0.0	0.0	0.0	41.3

^a^P value resulting from the comparison between the median values of bananas after eruption and control group (Mann-Whitney test).

^b^P value resulting from the comparison between the detection percentage (>LOD) of bananas after eruption and control group (Chi-square test).

n.s.: not significant.

#### 3.2.2. Potentially toxic elements.

High concentrations of potentially harmful elements, including aluminum, barium, and strontium, were found in both whole bananas and their pulp. (Table 3). Al levels increased during and immediately after the volcanic eruption, dropping by about tenfold within 8–12 months post-eruption (P < 0.001). This highlights the significant impact of recent volcanic materials on these banana cultivars and aligns with previous studies conducted in the aftermath of other volcanic eruptions, where aluminum accumulation was reported in both atmospheric and soil compartments. [62,63]. Such patterns have been attributed to the weathering of plagioclase-rich ash and mafic minerals, leading to enhanced Al^3^ ⁺ mobilization. This process is consistent with the observed dissolution of aluminosilicates in fresh basaltic deposits [70]. Ba and Sr, typically released from accessory minerals such as barite, feldspar, and Sr-bearing pyroxenes [71], showed elevated concentrations compared to controls but exhibited no clear temporal trends during or after the eruption (whole fruit: P = 0.031 and P < 0.001; pulp: P = 0.019 and P < 0.001). This pattern suggests slower release kinetics and stronger retention by soil colloids, particularly in young volcanic andosols. Evidence from water-rock interactions, altering elemental mobility, saturation indices, and cation ratios such as Ba^2^ ⁺ /Sr^2^ ⁺ supports this interpretation [71]. Redox conditions also play a crucial role: aerobic environments tend to enhance Ba and Sr mobility, while reducing conditions may promote their precipitation as sulfides or incorporation into secondary minerals [72].

**Table 3:** Analysis of potentially toxic element concentrations in whole bananas and banana pulp during and post-volcanic eruption. Data are expressed in ng/g fruit.

WHOLE BANANA																	
W	During eruption (n = 12)				At the end of eruption (n = 19)				After eruption (8–12 months) (n = 32)				Control group (n = 57)				Statistical significance
Element	Median	Upper limit	Lower limit	>LOD (%)	Median	Upper limit	Lower limit	>LOD (%)	Median	Upper limit	Lower limit	>LOD (%)	Median	Upper limit	Lower limit	>LOD (%)	*P*^*a*^/*P*^*B*^
Ag	0.0	0.1	0.0	58.3	0.0	0.1	0.0	65	0.0	0.1	0.0	27.2	0.0	0.1	0.0	41.3	n.s./n.s.
Al	5791.9	36244.4	2183.9	100	7461.1	13612.0	4951.0	100	784.1	1625.0	491.8	100	935.5	2404.5	218.4	95.7	<0.001/n.s.
Ba	214.5	444.3	101.6	100	189.7	398.4	100.2	100	111.0	142.6	50.0	100	113.7	176.2	33.4	100	0.031/n.s.
Be	0.0	0.0	0.0	16.7	0.0	0.1	0.0	30	0.0	0.3	0.0	42.4	0.0	0.0	0.0	26.1	n.s./n.s.
Cr	10.3	22.9	8.4	100	31.4	47.6	16.9	100	3.7	23.6	0.2	100	10.5	17.0	0.1	100	n.s./n.s.
Ni	32.3	49.2	15.2	100	36.4	52.3	27.8	100	33.8	43.3	22.4	100	21.4	46.5	11.8	100	0.011/n.s.
Pd	0.2	0.3	0.1	100	0.0	0.0	0.0	15	0.0	0.1	0.0	27.2	0.0	0.0	0.0	21.7	n.s./n.s.
Sb	0.1	0.1	0.0	66.7	0.0	0.0	0.0	10	0.0	0.0	0.0	21.2	0.0	0.0	0.0	23.9	n.s./n.s.
Sr	1150.1	1514.8	800.0	100	1136.2	1418.4	720.6	100	784.7	914.1	586.9	100	598.9	719.3	420.5	100	<0.001/n.s.
Th	0.5	4.1	0.1	91.7	0.6	1.5	0.4	100	0.0	0.5	0.0	33.3	0.1	0.1	0.0	63.1	n.s./0.009
Tl	0.4	0.9	0.2	100	0.8	1.6	0.5	80	0.0	4.7	0.0	48.5	0.0	1.6	0.0	45.7	n.s./n.s.
U	0.2	1.3	0.1	91.7	0.2	0.5	0.1	100	0.0	0.1	0.0	45.5	0.1	0.1	0.1	86.9	n.s./ < 0.000
V	12.9	91.0	2.9	100	18.6	45.5	10.6	100	2.6	4.4	1.7	100	1.0	1.4	0.8	100	<0.001/n.s.
**BANANA PULP**																	
Ag	0.0	0.0	0.0	41.7	0.0	0.1	0.0	55	0.0	0.0	0.0	21.2	0.0	0.0	0.0	39.1	n.s./n.s.
Al	1119.8	2414.8	519.5	100	2562.6	4611.0	1388.9	100	269.5	590.3	1.8	93.9	1.3	1.9	0.0	84.5	<0.001/n.s.
Ba	87.3	112.9	61.3	100	71.8	221.1	51.1	100	81.2	107.6	33.4	100	23.1	84.8	15.5	97.8	0.019/n.s.
Be	n.d.	n.d.	n.d.	n.d.	0.0	0.0	0.0	5	0.0	0.3	0.0	39.4	0.0	0.0	0.0	15.2	n.s./0.019
Cr	4.4	10.0	3.3	100	27.1	61.1	15.3	100	0.2	4.9	0.1	96.9	10.7	14.8	0.1	100	n.s/n.s.
Ni	27.5	44.6	18.6	100	44.8	69.5	31.3	100	47.0	58.4	28.9	100	22.1	34.9	12.9	100	<0.011/n.s.
Pd	0.1	0.1	0.0	75	0.0	0.0	0.0	5	0.0	0.0	0.0	3.1	0.0	0.0	0.0	2.1	n.s./n.s.
Sb	0.0	0.1	0.0	25	0.0	0.0	0.0	15	0.0	0.0	0.0	15.2	0.0	0.1	0.0	15.2	n.s./n.s.
Sr	494.8	555.5	430.1	100	457.8	606.1	389.3	100	431.5	537.4	352.3	100	245.5	306.5	206.2	100	<0.001/n.s.
Th	0.1	0.3	0.0	75	0.2	0.4	0.1	100	0.0	0.0	0.0	6.1	0.0	0.0	0.0	21.7	n.s./n.s.
Tl	0.3	0.8	0.2	100	0.7	1.4	0.0	65	0.0	1.8	0.0	39.4	0.0	1.8	0.0	43.4	n.s./n.s.
U	0.0	0.1	0.0	58.3	0.1	0.1	0.1	95	0.0	0.0	0.0	21.2	0.0	0.1	0.0	54.3	n.s./0.049
V	2.8	6.8	0.8	100	6.5	10.5	4.2	100	1.2	1.9	0.3	78.8	0.2	0.6	0.0	47.8	<0.001/0.009

^a^*P value* resulting from the comparison between the median values of bananas after eruption and control group (Mann-Whitney test).

^b^*P value* resulting from the comparison between the detection percentage (>LOD) of bananas after eruption and control group (Chi-square test).

n.s.: not significant; n.d.: not detected.

The eruption also influenced vanadium and nickel concentrations, minerals detected in 100% of the samples, with high V levels during the eruption compared to subsequent samples, especially in non-exposed regions (P < 0.001 in whole banana and pulp). This pattern may be explained by the ability of kaolinite and iron oxides to sequester vanadium in highly weathered soils through adsorption and structural incorporation, mechanisms especially relevant in iron oxide–rich environments under acidic conditions [73]. This is also reflected in a higher detection frequency in the edible part of the banana (P > 0.009). Ni concentrations in banana pulp samples post-eruption were significantly higher than in the control group (P < 0.001), indicating persistent environmental accumulation in the food chain over time. This trend may be associated with the presence of natural aluminosilicate nanoparticles, identified as effective complexing agents for nickel, which accounted for approximately 75% of the total Ni content in studied andosols and may consequently increase its bioavailability for plants such as banana.

**Fig 3:**
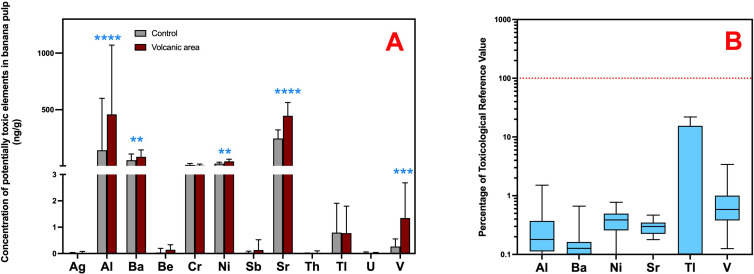
(A) Comparison of the potentially toxic elements content in banana pulp homogenates from samples collected 8–12 months after the volcanic eruption and from the control group. **P < 0.05; ****P < 0.001. (B) Box plots showing the percentage of the Toxicological Reference Value (Tolerable Daily Intake) of potentially toxic elements through the consumption of bananas from La Palma Island grown after the eruption period and influenced by volcanic emissions (8-12 months). The lines connect the medians, the boxes cover the 25th to 75th percentiles, and the minimal and maximal values are shown by the ends of the bars.

Taken together, the impact of Tajogaite’s volcanic materials must be considered, as significantly higher concentrations of Al, Ba, Ni, Sr, and V were observed in post-eruption samples compared to the control group. This effect is notable, given that the control group samples were primarily from Tenerife and Gran Canaria, more industrialized islands with higher population density, vehicular traffic, and intensified livestock and agricultural activities. Such anthropogenic factors are known contributors to the emission and accumulation of pollutants, including metals and toxic elements, in the environment [74]. On the other hand, there was limited influence of pyroclastic material and volcano-related fluxes on the presence of toxic elements such as beryllium, antimony, thorium, palladium, and uranium in the banana samples (Table 3). However, the volcano’s impact on the presence of Th, Tl, Pd, and U (samples>LOD) was evident during and at the final stage of the eruption, as their detection frequencies were lower in the samples analyzed after the eruption. Bananas are generally considered safe for consumption when evaluated for non-carcinogenic risks using the Tolerable Daily Intake guidelines. According to the present results, dietary intake constituting less than 1% of the hazardous threshold for continuous exposure to most examined elements in any scenario (Fig 3A and 3B). Thallium stands out as the sole element of potential concern, as high-percentile consumption can reach up to 22% of the TDI for Tl, significantly higher than the next highest element, vanadium, which accounts for only 3% of the TDI. This finding aligns with previous studies indicating that fruit consumption typically remains below the THQ for potentially harmful elements [68,75]. However, continued monitoring is particularly important, as studies have identified health risks for consumers of fruits and vegetables, such as nickel exceeding safe limits [61,76]. In this sense, the MOE value estimated for Ni in this study was 43.34 (>30), indicating a low health concern for systemic contact dermatitis associated with chronic exposure [33]. Interestingly, even though individual metal concentrations fall within safe limits, their presence can have unexpected effects on cells at exceedingly low doses. This phenomenon, known as non-linear cell response, suggests that the combined action of various metals may induce cellular damage at minimal levels, potentially contributing to the development of several diseases [63].

### 3.3. Effect of the volcanic eruption on the concentration and occurrence of REEs and MEs in Dietary intake and risk assessment

This study investigated the release of low-abundance crustal elements through volcanic activity, focusing on rare earth elements (REEs) and other microelements (MEs). To emphasize elements with a more significant presence in the banana samples, those with median or upper limit values below 0.2 ng/g in both the whole fruit and the pulp were excluded. These elements, including bismuth, osmium, platinum, ruthenium, lutetium, indium, and thulium, generally exhibit minimal presence and accumulation in food crops cultivated in the affected region, even after a volcanic episode.

Both whole fruit and pulp samples from the volcanic region showed notably higher concentrations, especially during and at the end of the eruption, compared to those from control areas (Table 4). Although REE levels in bananas from the same area decreased 8–12 months post-eruption, they remained elevated compared to non-exposed regions (ΣREEs = 4.2 and 2.0 ng/g, and 1.4 and 0.5 ng/g for whole banana and pulp, respectively; P < 0.001). Additionally, up to seven REEs (Ce, Dy, Er, Gd, La, Nd, Pr, and Y) were more frequently detected in samples exposed to magmatic conditions. These findings align with previous research demonstrating the presence of REEs in volcanic materials [13]. Consequently, these volcanic fluxes significantly influenced REEs content found in edible bananas, even though these elements are known to have low concentrations in environmental samples.

**Table 4:** Analysis of microelements (MEs) and rare earth elements (REEs) concentrations in whole bananas and banana pulp during and post-volcanic eruption. Data are expressed in ng/g fruit.

WHOLE BANANA																	
	During eruption (n = 12)				At the end of eruption (n = 19)				After eruption (8–12 months) (n = 32)				Control group (n = 57)				Statistical significance
Element	Median	Upper limit	Lower limit	>LOD (%)	Median	Upper limit	Lower limit	>LOD (%)	Median	Upper limit	Lower limit	>LOD (%)	Median	Upper limit	Lower limit	>LOD (%)	*P*^*a*^/*P*^*B*^
Au^d^	0.1	0.2	0.0	50	0.0	1.7	0.0	35	0.0	6.4	0.0	33.3	0.0	1.5	0.0	32.6	n.s./n.s
Ce^c^	11.8	84.4	2.5	100	11.5	33.9	7.6	100	1.5	2.3	0.9	96.9	0.7	1.1	0.2	76.1	<0.001./0.011
Dy^c^	0.4	3.3	0.1	91.7	0.5	1.3	0.3	100	0.1	0.2	0.0	66.7	0.0	0.1	0.0	56.5	0.035./n.s
Er^c^	0.2	1.4	0.0	83.3	0.2	0.6	0.1	95	0.0	0.1	0.0	45.5	0.0	0.0	0.0	45.7	n.s./n.s
Eu^c^	0.3	1.9	0.1	91.7	0.3	0.7	0.1	100	0.0	0.1	0.0	51.5	0.0	0.0	0.0	45.7	n.s./n.s
Ga^d^	2.1	16.7	0.5	100	1.8	4.6	1.4	100	0.3	0.6	0.2	93.9	0.2	0.3	0.1	82.6	<0.001/n.s
Gd^c^	0.7	5.1	0.1	91.7	0.7	1.8	0.4	100	0.1	0.2	0.1	90.9	0.1	0.1	0.0	71.7	<0.001./0.048
Ho^c^	0.1	0.6	0.0	75	0.1	0.2	0.1	90	0.0	0.0	0.0	27.2	0.0	0.0	0.0	19.6	n.s../n.s
La^c^	6.0	42.9	1.4	100	6.3	17.0	4.5	100	0.7	1.1	0.5	96.9	0.4	0.5	0.2	86.9	<0.001./n.s
Nb^d^	12.5	67.7	6.7	100	13.1	27.8	9.6	100	6.5	11.4	0.1	81.8	0.1	7.5	0.0	65.2	n.s./n.s
Nd^c^	4.9	35.8	1.0	100	5.2	14.2	3.2	100	0.7	1.0	0.4	93.9	0.3	0.4	0.2	82.6	<0.001./n.s
Pr^c^	1.4	10.1	0.3	100	1.4	3.9	0.9	100	0.2	0.3	0.1	90.9	0.1	0.1	0.0	73.9	<0.001./n.s
Sm^c^	0.9	6.2	0.2	100	0.9	2.5	0.6	100	0.1	0.2	0.0	69.7	0.1	0.1	0.0	71.7	0.031./n.s
Sn^d^	2.2	2.9	0.0	58.3	0.4	0.4	0.4	100	0.0	0.3	0.0	30.3	0.4	0.5	0.0	60.9	n.s./0.012
Ta^d^	6.1	9.3	3.4	100	3.6	8.6	2.3	100	2.4	7.1	0.0	81.8	0.1	3.6	0.0	73.9	n.s./n.s
Tb^c^	0.1	0.7	0.0	75	0.1	0.2	0.1	95	0.0	0.0	0.0	21.2	0.0	0.0	0.0	19.6	n.s./n.s
Ti^d^	979.1	7776.3	204.0	100	1245.0	3321.0	748.8	100	129.3	258.1	62.3	100	46.0	78.3	21.9	100	<0.001/n.s
Y^c^	2.2	16.6	0.5	100	2.3	6.1	1.7	100	0.4	0.7	0.2	96.9	0.2	0.3	0.2	91.3	<0.001/n.s
Yb^d^	0.1	1.1	0.0	75	0.1	0.4	0.1	95	0.0	0.1	0.0	33.3	0.0	0.0	0.0	43.5	n.s/n.s
Sum REEs	29.1	210.4	6.4	100	30.6	82.3	19.4	100	4.2	6.5	2.2	100	2.0	3.0	1.0	100	<0.001/n.s
**BANANA PULP**																	
Au^d^	0.0	0.2	0.0	33.3	0.0	0.9	0.0	25	0.0	0.0	0.0	9.1	0.0	0.0	0.0	19.6	n.s./n.s
Ce^c^	2.5	5.3	0.6	100	4.0	6.8	1.7	85	0.7	1.2	0.2	75.6	0.2	0.3	0.0	36.9	<0.001/ < 0.001
Dy^c^	0.1	0.2	0.0	83.3	0.2	0.3	0.1	100	0.0	0.1	0.0	36.4	0.0	0.0	0.0	8.7	0.048/0.004
Er^c^	0.0	0.1	0.0	58.3	0.1	0.1	0.0	95	0.0	0.0	0.0	18.2	0.0	0.0	0.0	2.2	n.s./0.019
Eu^c^	0.1	0.1	0.0	75	0.1	0.2	0.1	95	0.0	0.0	0.0	21.2	0.0	0.0	0.0	6.5	n.s./n.s
Ga^d^	0.5	1.1	0.1	100	0.8	1.1	0.4	100	0.0	0.3	0.0	48.5	0.0	0.1	0.0	39.1	n.s./n.s
Gd^c^	0.2	0.3	0.0	91.7	0.2	0.4	0.1	100	0.1	0.1	0.0	57.6	0.0	0.0	0.0	21.7	0.002/0.002
Ho^c^	0.0	0.0	0.0	33.3	0.0	0.1	0.0	50	0.0	0.0	0.0	3.1	0.0	0.0	0.0	4.3	n.s./n.s
La^c^	1.3	2.6	0.3	100	2.0	3.4	1.1	100	0.3	0.6	0.2	90.9	0.1	0.2	0.0	45.7	<0.001/ < 0.000
Nb^d^	5.6	10.1	3.2	100	5.8	10.7	3.8	100	0.1	0.1	0.0	69.7	0.1	0.1	0.0	54.3	n.s./n.s
Nd^c^	1.1	2.2	0.3	100	1.7	2.8	0.9	100	0.2	0.5	0.1	81.8	0.0	0.1	0.0	26.1	<0.001/ < 0.000
Pr^c^	0.3	0.6	0.1	100	0.5	0.8	0.2	100	0.0	0.1	0.0	45.5	0.0	0.1	0.0	19.6	n.s./0.024
Sm^c^	0.2	0.4	0.1	91.7	0.3	0.5	0.2	100	0.0	0.1	0.0	36.3	0.0	0.0	0.0	21.7	n.s./n.s
Sn^d^	0.0	2.5	0.0	41.7	0.4	0.4	0.4	100	0.0	0.3	0.0	33.3	0.0	0.0	0.0	60.1	0.032/0.022
Ta^d^	3.7	6.0	0.7	75	3.5	5.7	1.1	95	0.1	3.9	0.0	69.7	0.0	5.4	0.0	76.1	n.s./n.s
Tb^c^	0.0	0.0	0.0	33.3	0.0	0.1	0.0	65	n.d.	n.d.	n.d.	n.d.	0.0	0.0	0.0	4.3	n.s./n.s
Ti^d^	229.3	496.9	71.1	100	450.1	715.1	252.9	100	53.4	136.9	21.7	100	17.4	23.4	8.6	100	<0.001/n.s
Y^c^	0.5	1.0	0.2	100	1.0	1.5	0.5	100	0.2	0.4	0.0	66.7	0.1	0.1	0.0	41.3	0.022/0.039
Yb^d^	0.0	0.1	0.0	58.3	0.1	0.1	0.0	75	0.0	0.0	0.0	9.1	0.0	0.0	0.0	4.3	n.s./n.s
Sum REEs	6.3	13.0	1.6	100	9.8	17.1	4.5	100	1.4	3.0	0.5	100	0.5	0.8	0.1	100	<0.001/n.s

^a^*P value* resulting from the comparison between the median values of bananas after eruption and control group (Mann-Whitney test).

^b^*P value* resulting from the comparison between the detection percentage (>LOD) of bananas after eruption and control group (Chi-square test).

n.s.: not significant.

^c^RRE: Rare Eart Element; ^d^ME: other microelement.

Titanium concentrations were notably higher in the study samples during the volcanic eruption, gradually decreasing over time, while control samples consistently showed lower Ti levels, highlighting the significant influence of volcanic activity. These findings are consistent with data showing increased titanium deposition in soils under magmatic to magmatic-hydrothermal conditions [77]. Additionally, our analysis of REEs in the volcanic context revealed significant concentrations of neodymium and cerium, with both elements found at higher quantified levels in whole fruit samples compared to pulp. These Light REEs (Ce, La, and Nd), commonly hosted in labile phases of fresh volcanic glass, were disproportionately enriched in peel tissues, reflecting their preferential release during initial ash weathering [41]. In contrast, pulp samples exhibited higher Nd levels compared to Ce, indicating that root uptake and internal translocation reflect the speciation patterns and mobility of these elements in young andosols. The concentrations of these elements in bananas decreased significantly following the eruption (P < 0.001 in all cases, Table 4).

The volcanic eruption led to a prominent presence of several less environmentally abundant elements, including gallium, tantalum, lanthanum, and yttrium, in whole banana samples, underscoring how magmatic inputs can introduce new elemental fingerprints into plant systems. Notably, the concentration of La in whole fruit was six times higher than that found in the pulp, reflecting the accumulation of the volcanic ash on the peel. After the eruption, we observed lower concentrations of La, Nd, and Y in pulp samples. This trend becomes more evident when observing the detection of elements at each stage of the study, showing a gradual decline in their content in post-eruptive samples. This disparity diminished in subsequent sampling rounds, as ash‐derived REEs became progressively bound by soil colloids and organic matter. Consequently, total REEs concentrations were highest during the period of direct exposure to volcanic materials. In line with this, recent studies in similar volcanic environments demonstrate that freshly deposited soils display elevated REEs mobility, particularly within acidic microsites created by root and microbial secretions, before these elements are gradually immobilized in secondary mineral phases over months to years [78].

The present research suggests that volcanic eruptions release REEs and MEs into the environment, with eruption-related fluxes exerting a strong yet temporally limited influence on their accumulation in soils, plants, and banana fruits. Although concentrations declined after one year, the persistent elevation clearly indicates a lasting geochemical legacy in agricultural products. However, the risk associated with consuming these elements appears minimal. In the worst-case scenario (eating bananas harvested at the end of the eruption), the total REEs intake accounted for only 1.2% and 1.6% of the established daily limits for rare earth oxides or the sum of REEs, respectively. Nevertheless, caution is warranted when interpreting these results regarding the edible portion of bananas, given the potential health risks associated with REEs toxicity, such as oxidative stress and lipid peroxidation [44,45]. Further research is required to fully understand these effects and their potential impact on human health.

## 4. Limitations and strenghts of the study

The present study has several limitations that should be considered for proper interpretation of the results. First, the number of samples is limited due to accessibility constraints to the plantations in the regions exposed to the volcano. Second, no certified reference material was used for the banana analysis due to its non-existence commercially, although the recoveries of each element at each of the levels in the tailored matrices met the validation requirements in terms of accuracy, precision, repeatability, and reproducibility. Third, a more robust assessment of mineral element bioavailability and concentration would have been possible with the availability of pre-study baseline data from the investigated soils and bananas. Fourth, the sampling strategy and analytical procedures utilized in this study may have influenced the reported results. While efforts were made to ensure rigor, certain aspects may have impacted the observed outcomes. Finally, the opportunistic nature of the study, conducted in the aftermath of the Tajogaite Volcano eruption, resulted in various confounding factors not being accounted for in the present study, such as potential variations in soil type, moisture content, and topography, that could influence element uptake and transport.

However, despite these limitations, we believe that these data are highly relevant, as they allow us to visualize the influence of volcanic activity on the release of mineral elements into the environment and their subsequent incorporation into the food chain, with the resulting potential exposure of the population. Samples from the area affected by the volcano are of significant value due to the danger and difficult accessibility of the region after the eruption. The estimation of risks and benefits associated with banana consumption provides valuable baseline data for a wide range of elements, many of which are underrepresented in scientific literature, and supports future research on exposure to mineral elements from volcanic sources over time. This will not only strengthen the validity of our conclusions regarding the nutritional safety of bananas in the volcanic region but also provide a more robust basis for discussing their nutritional quality under volcanic stress conditions. Additionally, data are not only relevant to researchers but are also accessible to producers, consumers, and public health authorities. The availability of such data can inform strategies and measures to mitigate the impact of ecological disasters, while supporting efforts to protect public health and ensure food safety.

## 5. Conclusions

This study found significantly higher concentrations of both essential and non-essential elements in bananas from a volcanic area during and after the eruption, compared to samples from unaffected areas. In general, mineral concentrations decreased following the eruption. The increased presence of potentially toxic elements was linked to soil enrichment with pyroclastic material and ash. Furthermore, the elemental profile suggests minimal toxic risk, and despite variations, the nutritional value of bananas makes them a safe and healthy choice. Molybdenum and cobalt intake values reached up to 37% and 21% of the Recommended Daily Intake (RDI), respectively, which can be used as a dietary recommendation, especially for individuals with deficiencies in these elements. In contrast, for most toxic elements, the maximum estimated intake remained below 1% of the Tolerable Daily Intake (TDI). These findings contribute to the understanding of elemental composition in foodstuffs under volcanic exposure, providing valuable data on both essential nutrients and potential toxicological risks. In volcanic regions, continuous monitoring of contaminant levels in food crops is crucial to prevent human exposure to hazardous elements.

## Supporting information

S1 Table Limit of detection and quantification of the analysed elements (n = 50) expressed in ppb (µg/L). (DOCX)

S2 Table Unprocessed/raw data resulting from the analytical procedures. (XLSX)
