# Supplementary material for: Bananas in the aftermath of La Palma volcanic eruption (Canary Islands, Spain): A study on the nutritional and toxic element composition of post-disaster production
Source: PLoS One. 2025 Aug 11;20(8):e0328982. doi: 10.1371/journal.pone.0328982 (PMC12338782; doi:10.1371/journal.pone.0328982)
Supplement: S1 Table — (DOCX) [file pone.0328982.s001.docx]

**S1 Table.** Limit of detection and quantification of the analysed elements (n = 50) expressed in ppb (µg/L).

| **Symbol** | **Element** | **Limit of detection (LOD)** | **Limit of quantification (LOQ)** |
| --- | --- | --- | --- |
| **Essential Elements** |  |  |  |
| **Co** | Cobalt | 0,020 | 0,050 |
| **Cu** | Copper | 0,068 | 0,227 |
| **Fe** | Iron | 0,570 | 1,900 |
| **Mn** | Manganese | 0,036 | 0,120 |
| **Mo** | Molybdenum | 0,020 | 0,050 |
| **Se** | Selenium | 0,028 | 0,093 |
| **Zn** | Zinc | 0,035 | 0,117 |
| **Highly toxic elements** |  |  |  |
| **As** | Arsenic | 0,020 | 0,050 |
| **Cd** | Cadmium | 0,020 | 0,050 |
| **Hg** | Mercury | 0,026 | 0,087 |
| **Pb** | Lead | 0,059 | 0,197 |
| **Potentially toxic elements** |  |  |  |
| **Ag** | Silver | 0,020 | 0,050 |
| **Al** | Aluminium | 1,082 | 3,607 |
| **Ba** | Barium | 0,174 | 0,580 |
| **Be** | Beryllium | 0,020 | 0,050 |
| **Cr** | Chromium | 0,201 | 0,670 |
| **Ni** | Nickel | 0,167 | 0,557 |
| **Pd** | Palladium | 0,020 | 0,050 |
| **Sb** | Antimony | 0,020 | 0,050 |
| **Sr** | Strontitum | 0,057 | 0,190 |
| **Th** | Thorium | 0,020 | 0,050 |
| **Tl** | Thallium | 0,020 | 0,050 |
| **U** | Uranium | 0,020 | 0,050 |
| **V** | Vanadium | 0,020 | 0,050 |
| **Rare earth elements and other microelements** |  |  |  |
| **Au^b^** | Gold | 0,026 | 0,087 |
| **Bi^b^** | Bismuth | 0,020 | 0,050 |
| **Ce^a^** | Cerium | 0,020 | 0,050 |
| **Dy^a^** | Dysprosium | 0,020 | 0,050 |
| **Er^a^** | Erbium | 0,020 | 0,050 |
| **Eu^a^** | Europium | 0,020 | 0,050 |
| **Ga^b^** | Gallium | 0,020 | 0,050 |
| **Gd^a^** | Gadolinium | 0,020 | 0,050 |
| **Ho^a^** | Holmium | 0,020 | 0,050 |
| **In^b^** | Indium | 0,020 | 0,050 |
| **La^a^** | Lanthanum | 0,020 | 0,050 |
| **Lu^a^** | Lutetium | 0,020 | 0,050 |
| **Nb^b^** | Niobium | 0,076 | 0,253 |
| **Nd^a^** | Neodymium | 0,020 | 0,050 |
| **Os^b^** | Osmium | 0,020 | 0,050 |
| **Pr^a^** | Praseodymium | 0,020 | 0,050 |
| **Pt^b^** | Platinum | 0,020 | 0,050 |
| **Ru^b^** | Ruthenium | 0,020 | 0,050 |
| **Sm^a^** | Samarium | 0,020 | 0,050 |
| **Sn^b^** | Tin | 0,087 | 0,290 |
| **Ta^b^** | Tantalum | 0,072 | 0,240 |
| **Tb^a^** | Terbium | 0,020 | 0,050 |
| **Ti^b^** | Titanium | 0,097 | 0,323 |
| **Tm^a^** | Thulium | 0,020 | 0,050 |
| **Y^a^** | Yttrium | 0,020 | 0,050 |
| **Yb^a^** | Ytterbium | 0,020 | 0,050 |

**a: Rare Earth Element; b: Microelement.**
